# Immune-Related Gene Expression Responses to *In Ovo* Stimulation and LPS Challenge in Two Distinct Chicken Genotypes

**DOI:** 10.3390/genes15121585

**Published:** 2024-12-09

**Authors:** Anna Slawinska, Aleksandra Dunisławska, Artur Kowalczyk, Ewa Łukaszewicz, Maria Siwek

**Affiliations:** 1 Department of Animal Biotechnology and Genetics, Faculty of Animal Breeding and Biology, Bydgoszcz University of Science and Technology, St. Mazowiecka 28, 85-084 Bydgoszcz, Poland; 2 Department of Basic and Preclinical Sciences, Faculty of Biological and Veterinary Sciences, Nicolaus Copernicus University in Torun, Szosa Bydgoska 13, 87-100 Toruń, Poland; 3 Division of Poultry Breeding, Institute of Animal Husbandry and Breeding, Wroclaw University of Environmental and Life Sciences, Chełmońskiego 38C, 51-630 Wrocław, Poland; artur.kowalczyk@upwr.edu.pl (A.K.); ewa.lukaszewicz@upwr.edu.pl (E.Ł.)

**Keywords:** broilers, native chickens, systemic, mucosal immune responses, cytokine, gene expression

## Abstract

Background: *In ovo* stimulation introduces bioactive compounds, such as prebiotics, probiotics, or synbiotics into incubating eggs to enhance gut health and immune system development in chickens. This study aimed to determine the genetic and environmental effects modulating responses to *in ovo* stimulation in commercial broilers and Green-legged Partridge-like (GP) native chickens. Methods: Eggs were stimulated on day 12 of incubation with prebiotics (GOS—galactooligosaccharides), probiotics (*Lactococcus lactis* subsp. *cremoris*), or synbiotics (GOS + *L. lactis*), with controls being mock-injected. Hatched chicks were reared in group pens and challenged with lipopolysaccharide (LPS) on day 42 post-hatching. Cecal tonsils (CT) and spleens were harvested 2 h post-challenge. RT-qPCR was used to analyze the relative gene expression of cytokine genes: IL-1β, IL-2, IL-4, IL-6, IL-10, IL-12p40, and IL-17. Results: The results show that genotype influenced the expression of all immune-related genes, with broiler chickens exhibiting stronger innate inflammatory responses than native chickens. LPS induced both mucosal (CT) and systemic (spleen) immune responses in broilers but only systemic (spleen) responses in native chickens. Conclusions: *In ovo* stimulation had less of an impact on cytokine gene expression than LPS challenge. Broilers expressed higher inflammatory immune responses than GP native chickens.

## 1. Introduction

An avian model allows for a direct stimulation of the microbiota prior to hatching, by injecting bioactive compounds *in ovo* (into the egg). *In ovo*-delivered bioactive compounds (prebiotics, probiotics, and synergistic combination of both, i.e., synbiotics) stimulate intestinal microbiota, which primes the avian immune system [1]. The avian immune system is distinct from the mammalian one. The birds lack encapsulated lymph nodes, so the immune responses are mounted in peripheral immune organs, such as spleen, and diffuse lymphoid tissue, such as gut-associated lymphoid tissue (GALT) [2]. GALT is scattered along the entire intestinal tract. All GALT structures are formed during the embryonic phase but they develop post-hatching via the contact with antigens. Cecal tonsils (CT) are the largest structures in the chicken GALT. Ceca are the sites of the most diverse and abundant microbiota, which drive the CT maturation. CT consist of M cell-rich epithelium, sub-epithelial zone (populated predominantly with IgM+ and IgY+ B cells, and some CD4+ and CD8+ T cells), germinal centers (GC), and multiple follicles (CD4^+^ T cells). Phagocytic cells (macrophages and dendritic cells, DC) are present in the entire CT [2,3]. The spleen filters blood and triggers innate and adaptive immune responses to the antigens. Morphologically, it is composed of the white pulp, red pulp, and GC. These structures are built of both lymphoid (B and T cells) and non-lymphoid cells (macrophages and DC). Red pulp is composed of macrophages, plasma cells (producing immunoglobulins), and T cells (mostly CD8+, less CD4+). White pulp contains various populations of T cells (CD3+, CD4+, and CD8+), interdigitating DC, some macrophages, and B cells. Mature GC contain Bu-1+ B cells, a few T cells (CD3+ and CD4+), and follicular DC [2]. Both lymphoid organs, the CT and spleen, trigger innate and adaptive immune responses. CT sample intestinal antigens and trigger local (mucosal) immune responses, whereas the spleen is activated by the antigens sampled from blood.

The intensive selection of high-performing poultry resulted in the development of distinct genotypes with superior growth and feed efficiency (i.e., fast-growing meat-type poultry known as broilers) or egg-laying potential (i.e., egg-type chickens known as layers). Aside from commercially selected broilers and layers, there are dual-purpose breeds, which are slow-growing, native chickens used for meat and eggs production. Along with the distinct anatomy and physiology of broilers and layers, they differ in type and level of the immune responses. In fast-growing broilers, the immune responses are based on IgM antibodies, which represent short-term humoral immune responses [4]. In contrast, layers mount higher titers of IgY antibodies, representing long-term adaptive immune responses, combined with strong cellular immune responses [4]. Broilers are four-way crosses between heavily selected genetic lines. There is also a genetic component in the responses of the avian immune system to the intestinal microbiota. For example, the dextran sulfate sodium (DSS) administered to chickens early in life (days 11–18 post-hatching) disrupted intestinal homeostasis of both broilers and layers. But, the severity was lower in broilers than in layers, which was manifested by lower mortality and less damaged intestinal morphology [5]. Broilers treated with DSS, in contrast to layers, did not activate potent mucosal immune responses to inflammatory challenge. Their local humoral responses were based on increased IgM vs. IgY titers against LPS [5].

The strong selection pressure implemented in the contemporary broiler modulated not only growth and feed efficiency, but also the mechanisms of the immune responses. Cheema et al. (2003) compared the genetic line of a contemporary broiler from 2001 with a randombred broiler control from 1957, and determined differences in the immune parameters [6]. First, broilers from 1957 had greater relative weights of bursa of the Fabricius, CT and spleen in comparison to the contemporary broiler. This is a direct effect of the body weight increase due to selection, but without concurrent increases in the internal organs. Also, the randombred chickens from 1957 mounted higher humoral immune responses against sheep red blood cells in comparison with the broilers from 2001. On the other hand, contemporary broilers scored higher in the tests evaluating cellular and inflammatory immune responses [6]. In this study, we compared the immune-related gene expression in two distinct chicken genotypes. The fast-growing commercial broiler was analyzed in parallel with a light-weight, dual-purpose chicken (GP, Green-legged Partridgelike). GP is a Polish heritage chicken line, unselected since 1960s, and is currently kept in conservation flocks [7]. GP is an excellent model for immunological studies due to its conserved genetic status, as well as resilience and sturdiness.

Using the contrasting genetic makeup of the broilers and GP chickens, we have performed a study, in which we delivered a prebiotic, probiotic or synbiotic *in ovo* (on day 12 of eggs incubation), and tested various effects of such stimulation on the growing chickens. The early stimulation of the intestinal microbiota has a potent effect on the immune responses further in life [8], including responses to stress [9]. Transient immune responses can be triggered by Toll-like Receptor (TLR) ligands, such as lipopolysaccharide (LPS). LPS is an endotoxin produced by the Gram-negative bacteria and recognized TLR4 of the host animal [10]. Upon antigen recognition of the TLR ligand by the respective TLR, immune responses are generated in the peripheral immune organs and tissues, including mucosal and systemic genetic responses. The onset of the mucosal and systemic immune responses is manifested by cytokine secretion, including pro-inflammatory (IL-1β and IL-6), anti-inflammatory (IL-10), Th1 (IL-12), Th2 (IL-4), and Th17 (IL-17) cytokines [11,12].

Based on the above, we hypothesized that the distinct chicken genotypes would respond in a different manner to the *in ovo* stimulation of intestinal microbiota with bioactive compounds followed by immune challenge with LPS. The goals of the study were to (1) determine the local and systemic immune responses in chickens to pro-inflammatory antigens, and (2) estimate the effects of the host genetics and *in ovo* stimulation with prebiotics, probiotics, or synbiotics on the strength of the immune responses mounted upon challenge.

## 2. Results

### 2.1. Main Effects and Interactions

Table 1 presents the significance of the main effects (*in ovo* stimulation and LPS challenge) in the CT and spleen of two chicken genotypes (broiler chickens and native chickens). In broiler chickens, the majority of the genes were significantly influenced by LPS challenge, but not *in ovo* stimulation. This general pattern was determined for the CT as well as the spleen. The exceptions were determined for IL-2 and IL-4, for which both effects were insignificant in CT. Another exception was determined for IL-6 in the spleen, which was significantly influenced by both *in ovo* stimulation and LPS challenge.

In native GP chickens, the main effects were quite different. In CT, *in ovo* stimulation proved significant for the majority of the genes (except IL-1β and IL-6). However, LPS was significant only for IL-6 and IL-10. In the spleen, LPS was a main effect for the majority of the genes, except IL-4. Additionally, in the spleen, we found a significant effect of *in ovo* stimulation on IL-2, and two interactions between *in ovo* stimulation and LPS challenge for IL-4 and IL-12p40.

### 2.2. Immune-Related Gene Expression

#### 2.2.1. Broiler Chickens

**Figure 1:**
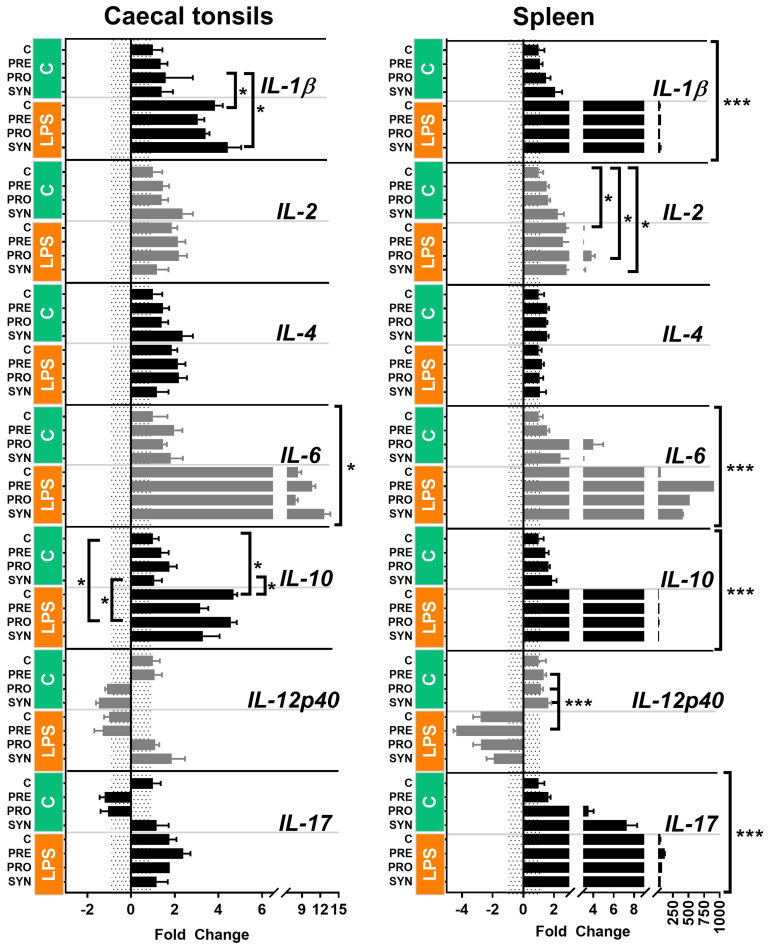
Gene expression responses to *in ovo* stimulation and LPS challenge determined in the CT and spleen of broiler chickens. *In ovo* stimulation was carried out on day 12 of egg incubation using three bioactive compounds: prebiotic (GOS—galactooligosaccharides), probiotic (*L. lactis*) and synbiotic (GOS + *L. lactis*). Controls were mock-injected. Hatched chicks were reared in the group pens. On day 42 post-hatching, immune challenge was applied (intraperitoneal injection of LPS—lipopolysaccharides). Controls were mock-injected. Samples of CT were harvested 2 h post-challenge for relative gene expression analysis of cytokine genes. Relative gene expression was performed using RT-qPCR and SYBR green chemistry. Cytokine genes *IL-1β*, *IL-2*, *IL-4*, *IL-6*, *IL-10*, *IL-12p40*, and *IL-17* were the target genes. *ACTB* and *UB* were used as reference genes. Calculations were based on the ΔΔCt method. Down-regulated data were transformed using the formula 2/-FC. Significance levels: *p* < 0.05 (*) and *p* < 0.001 (***). Graph prepared with GraphPad Prism 7 (GraphPad, La Jolla, CA, USA).

The dataset with the raw RT-qPCR data is included in Table S1. The relative cytokine gene expression is presented in Figure 1. The mRNA gene expression was more abundant in the spleen than in the CT. The majority of the genes were up-regulated in the LPS group. The most abundant mRNA gene expression in CT was determined for IL-6 (FC up to ~12 in the LPS group) and it was moderate for IL-1ß and *IL-10* (FC up to ~4 in LPS group). In the spleen, the gene expression was the most abundant for IL-1ß, IL-6, IL-10, and IL-17 genes (FC above 200 in LPS group).

#### 2.2.2. Native Chickens

**Figure 2:**
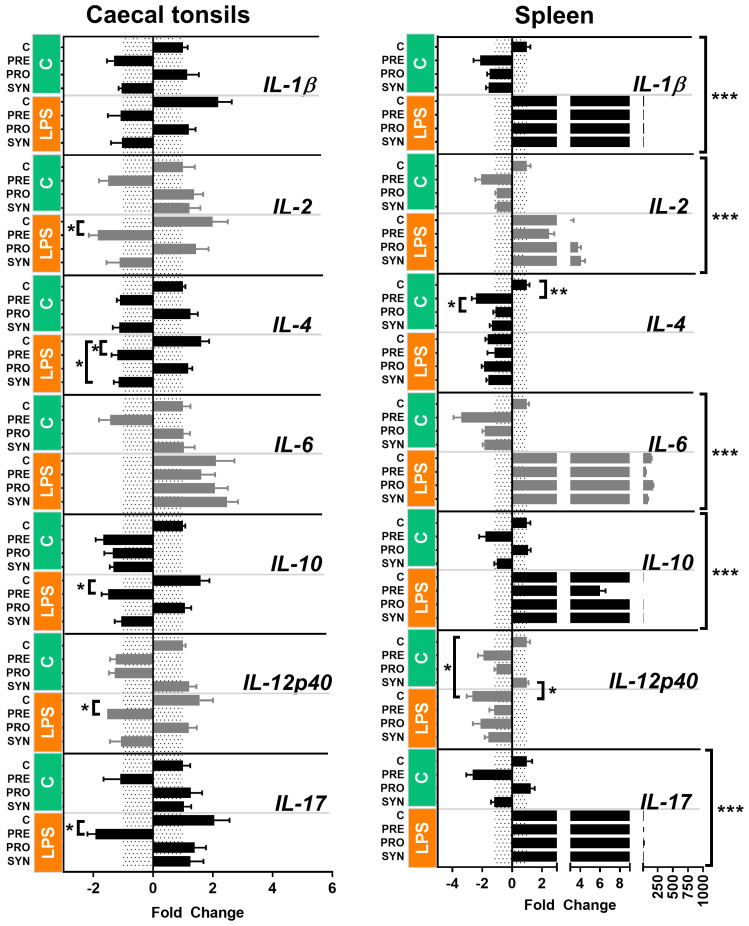
Gene expression responses to *in ovo* stimulation and LPS challenge determined in the CT and spleen of native chickens. *In ovo* stimulation was carried out on day 12 of egg incubation using three bioactive compounds: prebiotic (GOS—galactooligosaccharides), probiotic (*L. lactis*) and synbiotic (GOS + *L. lactis*). Controls were mock-injected. Hatched chicks were reared in group pens. On day 42 post-hatching, immune challenge was applied (intraperitoneal injection of LPS—lipopolysaccharides). Controls were mock-injected. Samples of CT were harvested 2 h post-challenge for the relative gene expression analysis of cytokine genes. Relative gene expression performed using RT-qPCR and SYBR green chemistry. Cytokine genes *IL-1β*, *IL-2*, *IL-4*, *IL-6*, *IL-10*, *IL-12p40*, and *IL-17* were target genes. *ACTB* and *UB* were used as reference genes. Calculatio were based on the ΔΔCt method. Down-regulated data were transformed using the formula 2/-FC. Significance levels: *p* < 0.05 (*), *p* < 0.01 (**) and *p* < 0.001 (***). Graph prepared with GraphPad Prism 7 (GraphPad, La Jolla, CA, USA).

The relative cytokine gene expression of native chickens is presented in Figure 2. The pattern of the cytokine gene expression was quite distinct in the CT of the native chickens. The mRNA abundance was rather low to moderate in all genes. We found some interesting differences between groups in post hoc tests, in which *in ovo* stimulation with a prebiotic (or synbiotic) induced the down-regulation of IL-2, IL-4, IL-10, IL-12p40, and IL-17 in LPS-challenged animals. The cytokine gene expression in the spleen resembled the same pattern as in broiler chickens. The mRNA abundance of the genes IL-1ß, IL-6, IL-10, and IL-17 was the highest in the LPS-stimulated individuals (FC above 200 in LPS group).

## 3. Discussion

### 3.1. Genetic Component of the Cytokine Gene Expression

In this study, we have tested the hypothesis that there are distinct mRNA gene expression responses between two contrasting chicken genotypes subjected to the same environmental stimulation. The statistical comparison between the results obtained for the two genotypes shows that the genotype is a factor influencing cytokine gene expression in broilers and native GP chickens (data not presented). The effect of the genetic component on the cytokine gene expression has been attributed to the accumulation of the genetic variation of the nucleotide sequences in the specific genes and their regulators [13]. To date, the largest genome-wide studies on the functional variation in cytokine production have been performed in humans [14]. This comprehensive study provides evidence that IL-6 cytokine relies on genetic variation to a greater extent than other cytokines. In chickens, the cytokine gene variation results from the adaptation to diverse environmental pressure and selective breeding [15]. For example, Mountford et al. (2022) found a large variation in the interferon signaling pathway between experimental chicken lines, which can be associated with the phenotypes that are resistant or susceptible to viral pathogens [13]. Zhou et al. (2020) studied the genomics of commercial broilers from 1957, 1978, and today [15]. Their results show that the selection pressure on the production traits also selects the genomic regions responsible for innate immune responses. In this case, the immune pathways were altered due to the variations that accumulated in the genes TLR3 and PLIN3 [15].

### 3.2. Immune-Related Aspect of In Ovo Stimulation

The effects of *in ovo* stimulation on the immune-related gene expression signatures in chickens are of particular interest. According to the review of Taha-Abdelaziz et al. (2018), the beneficial effects of early dietary interventions on the immune system in chickens manifest themselves in three areas: development of lymphoid organs, gastrointestinal microbiome, and immune competence [16]. Our previous and current research indicates that *in ovo*-delivered prebiotics, probiotics, and synbiotics affect all three aspects of poultry immunology [1].

Regarding the first aspect, which is lymphoid organs development, *in ovo* stimulation with prebiotics, probiotics and synbiotics significantly influenced the colonization of the spleen and CT with Bu-1+, CD4+, and CD8+ cells [8]. The influence depended on the bioactive compound, chicken genotype, and age. In broilers, *in ovo* stimulation increased CD4+ cells in CT (Day 7), CD4+ and CD8+ cells in CT and spleen (Day 21), as well as Bu-1+ cells in CT, and all types of lymphoid cells in the spleen (Day 42). In native chickens, synbiotics increased CD4+ and CD8+ cells in the spleen (but not the CT) (days 2, 21, and 42) [8].

Regarding the second aspect of early dietary interventions, i.e., gastrointestinal microbiome, to date we have demonstrated that the *in ovo* delivery of GOS prebiotics significantly increased the counts of lactobacilli and bifidobacteria in feces of newly hatched chicks [17]. These effects were long-lasting, and remained significant also on the day of slaughter. *Lactobacillus*-based synbiotics delivered *in ovo* on day 12 of egg incubation increased *Lactobacillus* spp. and *Enterococcus* spp. in the ileum of Cobb broiler chickens [18]. The current study reports that *in ovo* stimulation modulates immune competence (the third aspect of early dietary interventions) in broiler and native chickens.

### 3.3. Cytokine Gene Expression in Caecal Tonsils

The CT are the largest aggregates of lymphoid tissue present in chickens’ gut-associated lymphoid tissue (GALT). As such, they represent a major site of mucosal immune responses. In this study, an increased gene expression of *IL-1β* and *IL-6* in broiler chickens immunized with LPS reflects the acute inflammatory responses mounted by the GALT. The *IL-1β* and *IL-6* cytokines are produced primarily by macrophages and DC, and they are involved in acute inflammatory responses [19]. Both cytokines are activated by microbes, including enteric infection with *Eimeria* or *Salmonella* [11]. Haghighi et al. (2008) reported that the *IL-6* gene’s expression was increased in the CT of broiler chickens infected with *Salmonella*, but the inflammatory effects of infection were mitigated by probiotics [20]. In the current study, the broiler chickens immunized with LPS (also present in *Salmonella*) showed increased *IL-1β* and *IL-6* gene expression in the CT, but this was not influenced by *in ovo*-delivered bioactive compounds. In broiler chickens, *IL-10* cytokine expression was elevated by LPS. *IL-10* is an anti-inflammatory cytokine, and its expression counterbalances the pro-inflammatory activity of *IL-1β* and *IL-6* [21]. In other words, LPS-induced inflammation was regulated by negative feedback, which is the supposed mechanism of *IL-10* up-regulation in individuals expressing a high abundance of pro-inflammatory mediators (*IL-1β* and *IL-6*).

In native chickens, the results of the cytokine gene expression show that the *in ovo*-delivered bioactive compounds ameliorated LPS-induced inflammation by the down-regulation of some cytokines. The down-regulatory effect of GOS prebiotic (PRE) on *IL-12p40* and *IL-17* gene expression in the CT of native chickens is considered beneficial, since it helps in reducing LPS-induced inflammation in the intestines. Both cytokines are expressed by CD4+ cells (T lymphocytes) in response to inflammatory agents. The role of the IL-12p40 cytokine is to drive pro-inflammatory Th1-type responses, while pleiotropic cytokine IL-17 (also known as IL-17A) stimulates pro-inflammatory Th17-type responses [22]. Th17-type immune responses have recently been investigated due to their involvement in autoimmune diseases [23]. The increased number of Th17-type cells as well as IL-17 cytokine level eliminates the therapeutic effects of oral tolerance in mice [24]. The intestinal level of IL-17 varies depending on the microbiota composition. For example, *Lactobacillus fermentum* IM12 suppressed LPS-activated IL-17 level in mice [25]. On the other hand, dietary GOS increased IL-17 in the caecum of *Campylobacter*-infected broiler chickens, but did not ameliorate the infection [26]. We suppose that the decreased activity of *IL-17* gene expression in prebiotic-supplemented and LPS-challenged native chickens might be a good biomarker of the anti-inflammatory effects of prebiotics and probiotics delivered *in ovo*. However, more insight is required to determine the specific effects on the immune responses.

### 3.4. Cytokine Gene Expression in Spleen

The spleen is the largest peripheral immune organ in chickens, which filters blood and neutralizes the antigens of bacterial or viral origin. Since birds lack lymph nodes, the function of the spleen is crucial in mounting immune responses [27]. As such, the spleen is the site of the systemic immune responses to any antigen that is present in blood. In this study, LPS challenge was the main factor that influenced the gene expression of the cytokines in the spleen. In both genotypes, LPS challenge led to the up-regulated expression of pro-inflammatory *IL-1β* and *IL-6* cytokines (expressed primarily by macrophages and DC) as well as anti-inflammatory *IL-10* (expressed by the array of immune-related cells, including lymphoid and non-lymphoid cells), and regulatory *IL-17* cytokines (expressed primarily by Th17 cells, which are a subset of CD4+ lymphocytes). The difference between broilers and native chickens was seen in the level of up-regulation of the pro-inflammatory cytokines, which was higher in broiler chickens (e.g., relative *IL-6* gene expression in the LPS-prebiotic group was 900 FC in broiler chickens, but only 200 FC in native chickens). As mentioned earlier, the potent activation of the cellular and inflammatory immune responses is typical for the lymphoid system of the broiler chickens [6]. Broilers have been heavily selected for growth traits and feed efficiency [28]. Such strong selection pressure affected the immune systems of the broiler chickens. Acquired immune responses take weeks to develop functional antibodies against pathogens. Since a typical lifespan of a fast-growing broiler is only 35–42 days, strong innate immune responses are more able to fight infection [29]. The increased mRNA expression levels of pro-inflammatory cytokines are under genetic control, which may explain why, in this study, broilers mounted stronger inflammatory responses than native chickens.

The literature reports the beneficial effects of prebiotics, probiotics and other dietary supplements on ameliorating the inflammatory responses to LPS in chickens [30,31,32,33]. But, this study does not provide any evidence that *in ovo* stimulation with prebiotics, probiotics, or synbiotics alleviates LPS-induced inflammation in the chicken spleen. There have been recent studies linking gut microbiota and immunity in humans, which could not explain LPS-induced innate immune responses with reference to the existing variation in microbiota composition [34,35]. In particular, Habes et al. (2021) claimed that there are limitations in using microbiota as a modulator of the systemic innate immune responses of healthy humans to LPS challenge. Those results do not negate using an *in ovo* stimulation to improve the gut health of the chickens. The explanation of this phenomena may lay in the experimental approach. LPS is a universal antigen that triggers innate inflammatory responses, and we studied the acute effects on transient systemic immune responses in chickens. Perhaps, challenging with pathogens could show the potential for their exclusion by the properly primed microbiota of the host.

## 4. Materials and Methods

### 4.1. Experimental Design

**Figure 3:**
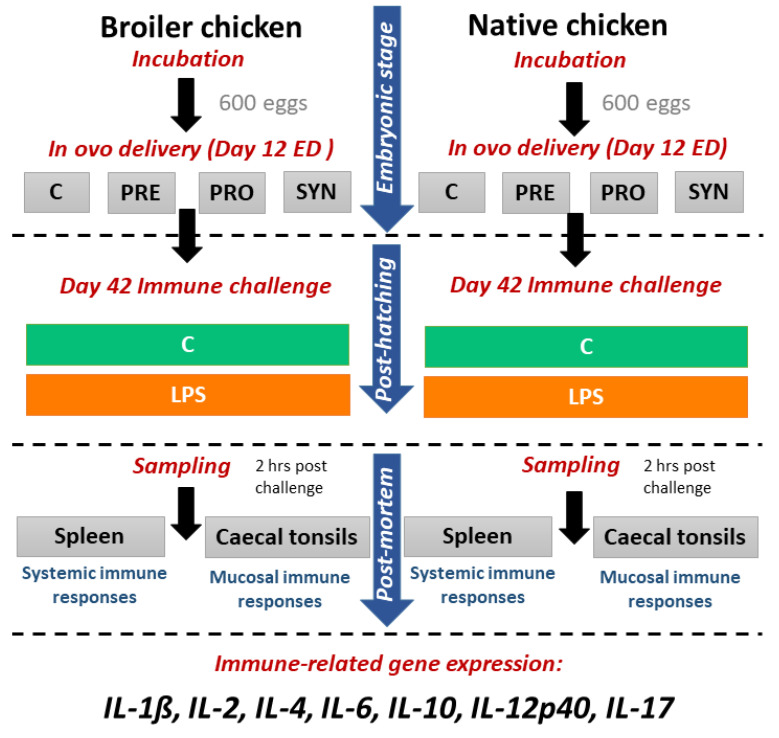
Experimental outline. The two trials were conducted on broiler and native chickens. The eggs were set in an incubator and on day 12 of incubation the bioactive compounds were injected into the air cell. C—physiological saline (control), PRE—galactooligosaccarides (3.5 mg/egg), PRO—*L. lactis* subsp. *cremoris* (10^5^ CFU/egg), SYN (combination of PRE and PRO). On day 42 of rearing, the chickens were immunized with lipopolysaccharide (LPS) or mock-immunized with physiological saline. Two hours post-immunization, all chickens were slaughtered and two tissues (CT and spleen) were harvested for immune-related gene expression analysis.

Trials were conducted on two chicken genotypes (broiler and native chicken). Each trial was based on the same full-factorial design, in which the experimental factors were as follows: (1) *in ovo* stimulation (with prebiotic, probiotic, or symbiotic vs. physiological saline), and (2) immune challenge with LPS vs. physiological saline. Figure 3 presents the experimental design described in this study. The treatments were followed by harvesting the CT and spleen to study local and systemic immune-related gene expression.

### 4.2. Animal Procedures

The experimental animals were broiler chicken (Ross 308, Aviagen) and native chicken (GP, Green-legged Partridgelike). Trials on both chickens started with egg incubation (600 eggs/genotype), followed by the *in ovo* injection of the respective bioactive compound on day 12 of incubation. The bioactive compounds for *in ovo* injection included prebiotics (GOS, galactooligosaccharides from Clasado Biosciences Ltd., Jersey, UK, 3.5 mg/egg), probiotics (*L. lactis* subsp. *cremoris* from IBB, PAS, Warsaw, Poland, 10^5^ CFU/egg), or synbiotics (GOS, 3.5 mg/egg + *L. lactis*, 10^5^ CFU/egg). Control eggs were mock-injected with sterile physiological saline. The injection volume for all eggs was 0.2 mL and the injection site was the air cell. After *in ovo* injection, the hole was sealed and the incubation continued until hatching.

After hatching, the chicks were housed in litter pens (4 replicates/group, 8 animals each) for 42 days. Feeding and environmental conditions were adjusted to the age and genotype of the birds. Commercial feed (TASOMIX, Biskupice Ołoboczne, Poland) and good-quality water were provided ad libitum. Table 2 gives an overview of the diets applied. The thermal and light conditions applied in the experiments for either chicken genotype are given in Supplementary Table S2. On the slaughter day, chickens were injected intraperitoneally with lipopolysaccharide (LPS, Sigma-Aldrich, Schnelldorf, Germany, cat.# L2880, 0.5 mg/kg body weight), or mock-injected with physiological saline. The LPS dose was selected based on data in the literature. Animals were sacrificed two hours post-injection and gene expression of the major immune mediators was assessed in the CT and spleen. Tissue samples (*n* = 8) were collected two hours after immune challenge. Samples of the spleen and CT were dissected and preserved in 3 mL fixRNA (EURx, Gdansk, Poland).

### 4.3. RNA Isolation and RT-qPCR

Total RNA was isolated from the CT and spleen. The tissues were first homogenized in 1 mL of the TRI Reagent (MRC, Cincinnati, OH, USA), using a rotor-stator homogenizer (TissueRuptor, Qiagen, GmbH, Hilden, Germany). The lysate was purified using a Universal RNA Purification Kit (EURx, Gdansk, Poland). The concentration and purity of the eluted RNA was measured with the NanoDrop (Thermo Scientific/NanoDrop Technologies, Wilmington, NC, USA). The total RNA was assessed for integrity by agarose gel electrophoresis.

Gene expression analysis was performed using 2-step RT-qPCR. An amount of 5 µg of total RNA was reverse-transcribed with the Maxima First Strand cDNA Synthesis Kit for RT-qPCR (Thermo Scientific, Vilnius, Lithuania). The obtained cDNA was diluted to a working concentration of 70 ng/µL and stored at −20 °C. RT-qPCR reactions were run using Maxima SYBR Green qPCR Master Mix (2x) (Thermo Scientific, Vilnius, Lithuania). The RT-qPCR reaction mix included 1x Maxima SYBR Green qPCR Master Mix, 1 µM of each oligonucleotide primer and 2 µL of diluted cDNA. The reaction volume was adjusted to 10 µL with nuclease-free water. Each RT-qPCR reaction was performed in two technical replicates. Sequences of the oligonucleotide primers used to amplify the immune-related genes are listed in Supplementary Table S3.

### 4.4. Statistical Analysis

#### 4.4.1. ANOVA

The experiment was performed according to a two-way factorial design, in which *in ovo* treatments (prebiotic vs. probiotic vs. synbiotic vs. control) and immune challenge (LPS vs. control) were considered independent variables, and the dCt of each target gene was a dependent variable. Statistical analysis was performed independently for each tissue (CT and spleen) and genotype (broiler and native chicken), using a two-way ANOVA with interactions. The main factors and their interactions were considered significant at *p* < 0.05, *p* < 0.01, or *p* < 0.001. The statistical analysis was performed using SAS Enterprise Guide 9.4 (SAS Institute, Cary, NC, USA).

#### 4.4.2. Relative Gene Expression

Relative gene expression analysis was performed based on Ct values from RT-qPCR data. The delta delta Ct (ddCt) algorithm was used to calculate the Fold Change (FC) of the gene expression in experimental groups in comparison to their respective controls [36]. The normalization of the mRNA expression of the target genes was performed with two reference genes (*ACTB* and *UB*). To calculate delta Ct (dCt), the mean Ct values of the two reference genes were subtracted from the Ct of each target gene (dCt = Ct target − Ct reference). The calibration of the relative gene expression was performed for each genotype/tissue independently. A calibrator was the C group (mock-injected *in ovo* and mock-immunized). The dCt value of the calibrator was subtracted from the dCt values of the experimental groups (ddCt = dCt experimental − dCt calibrator). FC was calculated with the following formula: FC = 2-ddCt. The ddCt calculations were performed in MS Excel and the FC values were visualized using GraphPad Prism 7 (GraphPad, La Jolla, CA, USA). The pairwise *t*-tests were performed within *in ovo*-treatment groups to indicate the significance of *in ovo* stimulation for the gene expression signatures (*p* < 0.05).

## 5. Conclusions

This study confirmed functional differences between immune responses in broiler and native chickens. Broiler chickens developed strong innate inflammatory immune responses to LPS in CT and spleen. Native GP chickens had lower inflammatory responses to LPS, but only in the spleen. Their mucosal immune responses were low and unaffected by LPS challenge. The *in ovo* stimulation had lower effects on the cytokine gene expression than LPS challenge. However, the GOS prebiotic delivered *in ovo* dampened the local immune responses in the CT in native chickens, even under LPS challenge. The results confirm that the genetic selection in broiler chickens clearly increased their inflammatory responses, while the native chickens have developed more tolerant mucosal immune resposnes.

## Figures and Tables

**Table 1:** The effects of immune challenge, in ovo stimulation and their interaction on the immune-related gene expression signatures in cecal tonsil and spleen of broilers and native chickens *.

**Broiler Chickens**							
**Cecal Tonsil**				**Spleen**			
**Gene**	***In Ovo***	**LPS**	***In Ovo ×* LPS**		***In Ovo***	**LPS**	***In Ovo ×* LPS**
IL-1β	NS	<0.001	NS		NS	<0.001	NS
IL-2	NS	NS	NS		NS	<0.001	NS
IL-4	NS	NS	NS		NS	<0.05	NS
IL-6	NS	<0.001	NS		<0.01	<0.001	NS
IL-10	NS	<0.001	NS		NS	<0.001	NS
IL-12p40	NS	<0.05	NS		NS	<0.001	NS
IL-17	NS	<0.01	NS		NS	<0.001	NS
**Native Chickens**							
**Cecal ToNSil**				**Spleen**			
**Gene**	***In Ovo***	**LPS**	***In Ovo ×* LPS**		***In Ovo***	**LPS**	***In Ovo ×* LPS**
IL-1β	NS	NS	NS		NS	<0.001	NS
IL-2	<0.01	NS	NS		<0.05	<0.001	NS
IL-4	<0.01	NS	NS		NS	NS	<0.001
IL-6	NS	<0.001	NS		NS	<0.001	NS
IL-10	<0.001	<0.05	NS		NS	<0.001	NS
IL-12p40	<0.05	NS	NS		NS	<0.01	<0.01
IL-17	<0.05	NS	NS		NS	<0.001	NS

* Effects: *In ovo* stimulation with prebiotic (GOS—galactooligosaccharides), probiotic (*L. lactis*) or synbiotic (GOS + *L. lactis*) vs. physiological saline (C) on day 12 of egg incubation. Immune challenge with lipopolysaccharide (LPS) by intraperitoneal injection on day 42 post-hatching; interaction between LPS challenge and *in ovo* stimulation. Gene expression analysis was performed with RT-qPCR. The significance of effects was calculated with two-way ANOVA. Significance levels: *p* < 0.05, *p* < 0.01 or *p* < 0.001 (significant), and *p* > 0.05 (non-significant, NS).

**Table 2:** Chemical composition of commercial feeds used for chicken broilers and native chickens.

Items	Broiler Chickens				Native Chickens	
	Starter (D 1-10)	Grower I (D 11-21)	Grower II (D 22-33)	Finisher (D 34-42)	Starter (D 1-28)	Grower (D 19-42)
MEN (MJ/kg)	12.50	12.95	13.35	13.41	11.9	11.7
Crude protein (g/kg)	220	200	190	184	200	185
Crude fiber (g/kg)	28.00	30.0	31.0	32.0	34.0	35.0
Lysine (g/kg)	13.8	12.5	11.3	10.5	11.0	10.0
Methionine + Cystine (g/kg)	10.3	9.5	8.8	8.2	8.2	7.2
Threonine (g/kg)	9.2	8.3	7.6	7.2	7.6	7.0
Tryptophan (g/kg)	2.2	2.0	1.9	1.9	2.1	2.0

## Data Availability

The datasets used in the current study are available from the corresponding author on reasonable request.

## Supplementary Materials

The following supporting information can be downloaded at: https://www.mdpi.com/article/10.3390/genes15121585/s1, Table S1: Raw RT-qPCR data; Table S2: Environmental conditions used for chicken broilers and native GP chicken, and Table S3: Immune-related genes and primers for RT-qPCR analysis.
