# Supplementary material for: Immune-Related Gene Expression Responses to In Ovo Stimulation and LPS Challenge in Two Distinct Chicken Genotypes
Source: Genes (Basel). 2024 Dec 9;15(12):1585. doi: 10.3390/genes15121585 (PMC11675432; doi:10.3390/genes15121585)
Supplement: Supplementary file 1 [file genes-15-01585-s001.zip › genes-3307352-supplementary/S2 Table.pdf]

S2 Table. Environmental conditions used for chicken broilers and native GP chicken

<table>
<tr><th rowspan="2">Age (days)</th><th colspan="2">Temperature (°C)</th><th>Relative humidity (%)</th><th colspan="2">Lighting program (light/dark)</th><th colspan="2">Light intensity (lux)</th></tr>
<tr><th>broiler</th><th>native</th><th></th><th>broiler</th><th>native</th><th>broiler</th><th>native</th></tr>
<tr><td>1</td><td>31</td><td>32</td><td rowspan="12">60-65</td><td>24/0</td><td rowspan="2">22/2</td><td rowspan="3">40</td><td rowspan="3">30</td></tr>
<tr><td>3</td><td>29</td><td>31</td><td>23/1</td></tr>
<tr><td>6</td><td>28</td><td>30</td><td>22/2</td><td>20/4</td></tr>
<tr><td>9</td><td>27</td><td rowspan="2">28 - 26</td><td rowspan="9">18/6</td><td rowspan="3">19/5</td><td rowspan="9">20</td><td rowspan="3">20</td></tr>
<tr><td>12</td><td>26</td></tr>
<tr><td>15</td><td>25</td><td rowspan="3">26 - 24</td></tr>
<tr><td>18</td><td>24</td><td rowspan="4">18/6</td><td rowspan="6">10</td></tr>
<tr><td>21</td><td>23</td></tr>
<tr><td>24</td><td>22</td><td rowspan="2">24 - 20</td></tr>
<tr><td>27</td><td>21</td></tr>
<tr><td>35</td><td>20</td><td rowspan="2">20 - 18</td><td>17/7</td></tr>
<tr><td>36-42</td><td>18</td><td>16/8</td></tr>
</table>
