# Supplementary material for: Immune-Related Gene Expression Responses to In Ovo Stimulation and LPS Challenge in Two Distinct Chicken Genotypes
Source: Genes (Basel). 2024 Dec 9;15(12):1585. doi: 10.3390/genes15121585 (PMC11675432; doi:10.3390/genes15121585)
Supplement: Supplementary file 1 [file genes-15-01585-s001.zip › genes-3307352-supplementary/S3 Table.pdf]

S3 Table. Immune-related genes and primers for RT-qPCR analysis

Gene symbol	Gene ID	Primer sequence (5′→3′)	Reference
		Target genes	
IL-1β	395196	F: GGAGGTTTTTGAGCCCGTC<br>R: TCGAAGATGTCGAAGGACTG	[1]
IL-2	373958	F: GCTTATGGAGCATCTCTATCATCA<br>R: GGTGCACTCCTGGGTCTC	[2]
IL-4	416330	F: GCTCTCAGTGCCGCTGATG<br>R: GGAAACCTCTCCCTGGATGTC	[3]
IL-6	395337	F: AGGACGAGATGTGCAAGAAGTTC<br>R: TTGGGCAGGTTGAGGTTGTT	[4]
IL-10	428264	F: CATGCTGCTGGGCCTGAA<br>R: CGTCTCCTTGATCTGCTTGATG	[5]
IL-12p40	404671	F: TTGCCGAAGAGCACCAGCCG<br>R: CGGTGTGCTCCAGGTCTTGGG	[6]
IL-17	395111	F: GGGATTACAGGATCGATGAGGA<br>R: GAGTTCACGCACCTGGAATG	[2]
		Reference genes	
ACTB	396526	F: CACAGATCATGTTTGAGACCTT<br>R: CATCACAATACCAGTGGTACG	[7]
UB	396190	F: GGGATGCAGATCTTCGTGAAA<br>R: CTTGCCAGCAAAGATCAACCTT	[7]

Reference list:

1. Dunislawska, A.; Slawinska, A.; Stadnicka, K.; Bednarczyk, M.; Gulewicz, P.; Jozefiak, D.; Siwek, M. Synbiotics for Broiler Chickens—In Vitro Design and Evaluation of the Influence on Host and Selected Microbiota Populations Following In Ovo Delivery. *PLOS ONE* **2017**, *12*, e0168587, doi:10.1371/journal.pone.0168587.
2. Pietrzak, E.; Dunislawska, A.; Siwek, M.; Zampiga, M.; Sirri, F.; Meluzzi, A.; Tavaniello, S.; Maiorano, G.; Slawinska, A. Splenic Gene Expression Signatures in Slow-Growing Chickens Stimulated in Ovo with Galactooligosaccharides and Challenged with Heat. *Animals (Basel)* **2020**, *10*, 474, doi:10.3390/ani10030474.
3. Sławinska, A.; Siwek, M.Z.; Bednarczyk, M.F. Effects of Synbiotics Injected in Ovo on Regulation of Immune-Related Gene Expression in Adult Chickens. *Am J Vet Res* **2014**, *75*, 997–1003, doi:10.2460/ajvr.75.11.997.
4. Chiang, H.-I.; Berghman, L.R.; Zhou, H. Inhibition of NF-kB 1 (NF-kBp50) by RNA Interference in Chicken Macrophage HD11 Cell Line Challenged with Salmonellaenteritidis. *Genet Mol Biol* **2009**, *32*, 507–515, doi:10.1590/S1415-47572009000300013.
5. Rothwell, L.; Young, J.R.; Zoorob, R.; Whittaker, C.A.; Hesketh, P.; Archer, A.; Smith, A.L.; Kaiser, P. Cloning and Characterization of Chicken IL-10 and Its Role in the Immune Response to Eimeria Maxima. *J Immunol* **2004**, *173*, 2675–2682, doi:10.4049/jimmunol.173.4.2675.
6. Brisbin, J.T.; Gong, J.; Parvizi, P.; Sharif, S. Effects of Lactobacilli on Cytokine Expression by Chicken Spleen and Cecal Tonsil Cells. *Clin Vaccine Immunol* **2010**, *17*, 1337–1343, doi:10.1128/CVI.00143-10.
7. De Boever, S.; Vangestel, C.; De Backer, P.; Croubels, S.; Sys, S.U. Identification and Validation of Housekeeping Genes as Internal Control for Gene Expression in an Intravenous LPS Inflammation Model in Chickens. *Vet Immunol Immunopathol* **2008**, *122*, 312–317, doi:10.1016/j.vetimm.2007.12.002.
